# Dysregulated Autophagy and Mitophagy in a Mouse Model of Duchenne Muscular Dystrophy Remain Unchanged Following Heme Oxygenase-1 Knockout

**DOI:** 10.3390/ijms23010470

**Published:** 2021-12-31

**Authors:** Olga Mucha, Katarzyna Kaziród, Paulina Podkalicka, Kinga Rusin, Józef Dulak, Agnieszka Łoboda

**Affiliations:** Department of Medical Biotechnology, Faculty of Biochemistry, Biophysics and Biotechnology, Jagiellonian University, 30-387 Krakow, Poland; olga.mucha@doctoral.uj.edu.pl (O.M.); katarzyna.kazirod@doctoral.uj.edu.pl (K.K.); paulina.podkalicka@doctoral.uj.edu.pl (P.P.); kinga.rusin@selvita.com (K.R.); jozef.dulak@uj.edu.pl (J.D.)

**Keywords:** DMD, Duchenne muscular dystrophy, mitophagy, autophagy, *mdx*, heme oxygenase-1, HO-1

## Abstract

Dysregulation of autophagy may contribute to the progression of various muscle diseases, including Duchenne muscular dystrophy (DMD). Heme oxygenase-1 (HO-1, encoded by *Hmox1*), a heme-degrading enzyme, may alleviate symptoms of DMD, inter alia, through anti-inflammatory properties. In the present study, we determined the role of HO-1 in the regulation of autophagy and mitophagy in *mdx* animals, a commonly used mouse model of the disease. In the gastrocnemius of 6-week-old DMD mice, the mRNA level of mitophagy markers: *Bnip3* and *Pink1*, as well as autophagy regulators, e.g., *Becn1*, *Map1lc3b*, *Sqstm1*, and *Atg7*, was decreased. In the dystrophic diaphragm, changes in the latter were less prominent. In older, 12-week-old dystrophic mice, diminished expressions of *Pink1* and *Sqstm1* with upregulation of *Atg5*, *Atg7*, and *Lamp1* was depicted. Interestingly, we demonstrated higher protein levels of autophagy regulator, LC3, in dystrophic muscles. Although the lack of *Hmox1* in *mdx* mice influenced blood cell count and the abundance of profibrotic proteins, no striking differences in mRNA and protein levels of autophagy and mitophagy markers were found. In conclusion, we demonstrated complex, tissue, and age-dependent dysregulation of mitophagic and autophagic markers in DMD mice, which are not affected by the additional lack of *Hmox1*.

## 1. Introduction

Skeletal muscles, characterized by high metabolic activity [1] and constantly exposed to mechanical and oxidative stress, are particularly susceptible to the formation of dysfunctional organelles and protein aggregates [2]. Whether in response to the stressor cells decide to undergo apoptosis or autophagy, is strictly dependent on the nature of the stimulus and the balance between inhibitory and activating signals [3,4]. The sustained excessive level of autophagy flux degrades a large number of vital molecules and organelles, while not sufficient autophagic machinery causes protein inclusions and accumulation of damaged organelles [1,5]. Therefore, autophagy plays a pivotal role in the proper functioning and repair of the muscle [6,7].

A growing number of studies indicate dysregulation of autophagic flux in myoblasts as a hallmark of numerous muscular diseases, including muscular dystrophies [8]. In Duchenne muscular dystrophy (DMD), the disease caused by a lack of dystrophin, a protein essential for myocyte integrity, dysfunctional autophagy was demonstrated to contribute to muscle weakness and wasting [9,10,11,12]. Furthermore, systemic metabolic impairment, including disruption in Ca^2+^ homeostasis, augmented reactive oxygen species (ROS) production, and mitochondrial dysfunction, greatly contribute to the progression of this incurable disorder (reviewed in: [13]). Therefore, autophagy and organelle-specific autophagy, namely mitophagy, may be of particular importance in DMD [11,12].

Accordingly, normalization of defective autophagy/mitophagy was suggested as an experimental therapy for DMD. Resveratrol, a polyphenol found in grapes and red wine, led to mitophagy stabilization, reduction of ROS levels, and improvement of muscle condition in *mdx* animals, a mouse model of DMD [11]. Kuno et al. demonstrated that treatment of dystrophic mice with this polyphenol for 56 weeks ameliorates mitophagy and decreases cardiomyopathy, the life-threatening heart complication during DMD progression [8]. Interestingly, Whitehead et al. noted that the use of simvastatin (an HMG-CoA reductase inhibitor, generally used to normalize cholesterol levels) has a positive effect on autophagy in the DMD mouse model [14,15]. Recently, restoration of functional autophagy in dystrophic skeletal muscle was also observed after treatment with a hydrogen sulfide donor [16]. Of note, it was also underlined that expression of mitophagy- and autophagy-related proteins may vary during mice lifetime [17].

An important aspect of maintaining homeostasis in skeletal muscles is counteracting oxidative stress. In DMD, sustained oxidative stress aggravates muscle damage and contributes to disease progression, hence the use of antioxidants may be of therapeutic importance [18]. One of the candidates might be heme oxygenase-1 (HO-1, encoded by *Hmox1*), a cytoprotective enzyme that through heme degradation generates anti-inflammatory end products (mostly CO) with protective roles in physiological and pathological conditions. In our previous study, chemical inhibition of HO-1 activity as well as global, genetic knockout of *Hmox1* in dystrophic animals, increased the inflammatory reaction, deteriorated the condition of the muscles, and decreased mice endurance to exercise [19]. HO-1 was also demonstrated to control the activation of autophagy to counteract stress conditions and inflammation in various diseases, including sepsis, diabetes, or reperfusion after hypoxia in the liver (reviewed in: [20]). Moreover, the appearance of abnormal mitochondria together with impaired Pink1/Parkin-dependent mitophagy and mitochondrial biogenesis were noticed in *Hmox1*-lacking animals (reviewed in: [21]). Of note, to the best of our knowledge, no data regarding the possible role of HO-1 in the control of the autophagy or mitophagy pathways in DMD has been published.

Therefore, we aimed to investigate the role of HO-1 in dysregulated autophagy and mitophagy in *mdx* mice. The mitochondrial dysfunction in dystrophic animals was evident at the mRNA and protein level in the diaphragm and gastrocnemius muscles. Although a decrease in the expression of genes regulating mostly mitophagy was observed in 6-week-old *mdx* mice, in double knockout animals–lacking dystrophin and *Hmox1* (*mdx/Hmox1^−/−^*), no changes were observed, questioning our hypothesis about the beneficial effect of this cytoprotective enzyme in this process. However, at the protein level, the expression of autophagy markers was increased by dystrophin deficiency, indicating additional regulation at the translational level. As we observed muscle and age-dependent effects, more research is still required to better understand molecular mechanisms of “self-eating” as well as HO-1 role in the autophagy dysregulation seen in DMD (especially in experimental settings with HO-1 overproduction/overexpression), where it could be a potential therapeutic agent.

## 2. Results

### 2.1. Verification of the Animal Models Used in the Study

**Figure 1:**
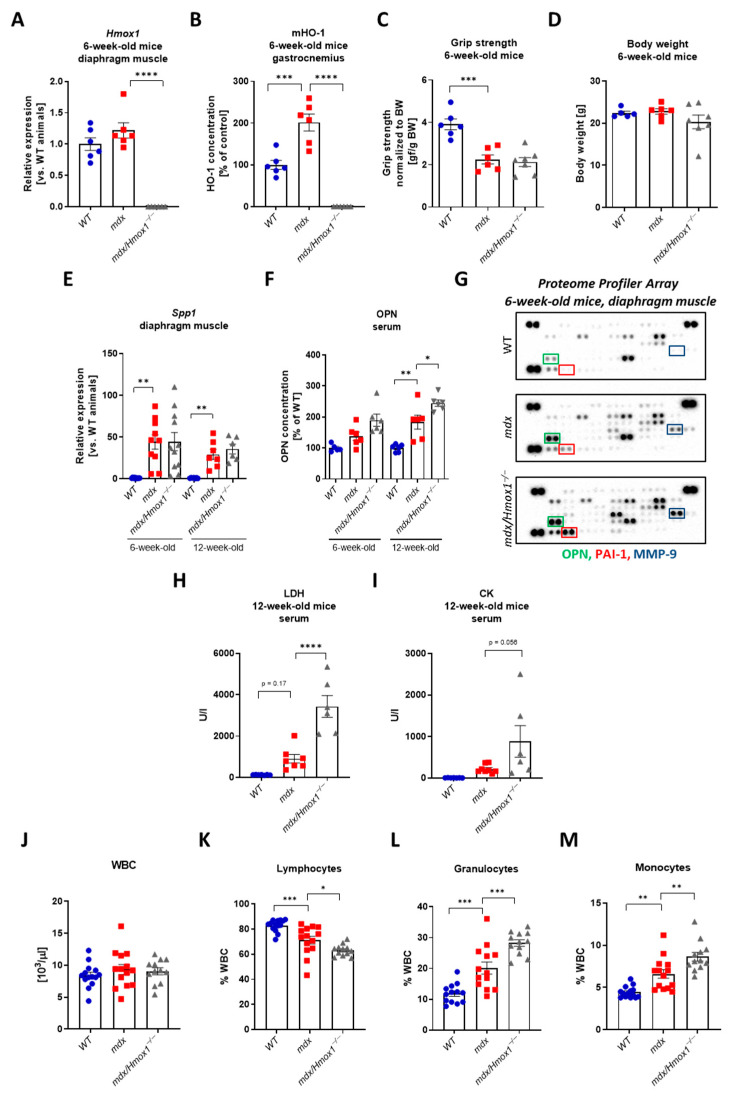
Validation of the animal model. *Hmox1* expression in 6-week-old animals (**A**), qRT-PCR; n = 6/group. The protein level of HO-1 in gastrocnemius muscle (**B**), ELISA, n = 6. Dystrophic mice exhibit decreased muscular strength (**C**)**,** grip test, n = 6–7/group; and no difference in body weight (**D**), n = 5–7/group. The expression of DMD markers: *Spp1* in the diaphragm (**E**)**,** qRT-PCR; n = 10–11/group and its protein level (osteopontin, OPN) in the serum of 6- and 12-week-old mice (**F**), ELISA; n = 5–6/group. The expression of OPN, matrix metalloproteinase-9 (MMP-9), and plasminogen activator inhibitor-1 (PAI-1) in the diaphragm of 6-week-old mice (**G**), Proteome Profiler™ array, representative results. Serum level of lactate dehydrogenase, LDH (**H**) and creatine kinase, CK (**I**) activity measured in 12-week-old mice; n = 6–8/group. The number of white blood cells (WBC) (**J**) and the percentage of lymphocytes (**K**), granulocytes (**L**), and monocytes (**M**); blood cell count, n = 12–14/group; Results presented as mean ± SEM. * *p* < 0.05, ** *p* < 0.01, *** *p* < 0.005, **** *p* < 0.0001 by one-way ANOVA with Tukey’s post hoc test.

Although mdx mice, with a nonsense point mutation (C-to-T transition) in exon 23 that aborts full-length dystrophin expression [22], are not severely affected (as compared to humans), and the differences in the disease outcomes between skeletal muscles and the diaphragm are known [23], they are commonly employed model animals in DMD research. To investigate the role of HO-1 in DMD, we generated *mdx* mice lacking the *Hmox1* gene, encoding HO-1, (named as *mdx*/*Hmox1^−/−^*), as described in our previous study [19]. In such double knockouts, expression of *Hmox1* was not detectable, and it did not differ between 6-week-old wild-type (WT) and *mdx* animals (Figure 1A).

On the protein level, the lack of HO-1 in dystrophic animals was also confirmed, together with potent upregulation of its level in *mdx* vs. WT mice (Figure 1B). In dystrophic mice, we detected decreased muscle strength (Figure 1C) without any differences in body weight (Figure 1D) in comparison to WT counterparts. Both parameters were not affected by the lack of HO-1 (Figure 1C,D).

Further analysis confirmed the DMD phenotype, as the upregulated level of *Spp1* encoding osteopontin (OPN), the fibrotic and inflammatory factor [24,25], was detected in the diaphragm of 6-week-old *mdx* mice and it was persistently elevated in 12-week-old animals (Figure 1E). The protein level of this DMD marker was upregulated in the serum of older (Figure 1F) dystrophic mice.

However, no differences were found in *Spp1* expression in the diaphragm of 6- and 12-week-old *mdx*/*Hmox1^−/−^* animals in comparison to *mdx* mice (Figure 1E). In serum, its protein level was higher in mice lacking *Hmox1* only in older animals (Figure 1F). A similar pattern was found in proteome profiler array analysis as OPN was one of the highly upregulated proteins in dystrophic animals (Figure 1G). Moreover, the level of factors regulating extracellular matrix remodeling, affecting muscle regeneration like plasminogen activator inhibitor-1 (PAI-1) and matrix metalloproteinase-9 (MMP-9) [26], was potently increased in the diaphragm of 6-week-old *mdx* mice (Figure 1G). Lack of *Hmox1* accelerated the expression of the abovementioned factors on the protein level (Figure 1G) suggesting aggravated phenotype of the disease. In older animals, as in our previous work [19], HO-1 deficiency resulted in the elevated activity of lactate dehydrogenase (LDH) (Figure 1H) and creatine kinase (CK) (Figure 1I), the indicators of muscle damage, already increased in dystrophic animals. Furthermore, although the mice of the three genotypes did not exert any differences in the number of white blood cells (WBC) (Figure 1J), the percentage of lymphocytes among WBC was decreased in dystrophic animals and was further diminished in *mdx*/*Hmox1*^−/−^ mice (Figure 1K). Oppositely, a higher percentage of granulocytes and monocytes was found in *mdx* mice and was even further increased in double knockouts (Figure 1L,M).

### 2.2. The Expression of Genes Modulating Autophagy Is Selectively Regulated in Gastrocnemius and the Diaphragm of 6-Week-Old Dystrophic Mice

**Figure 2:**
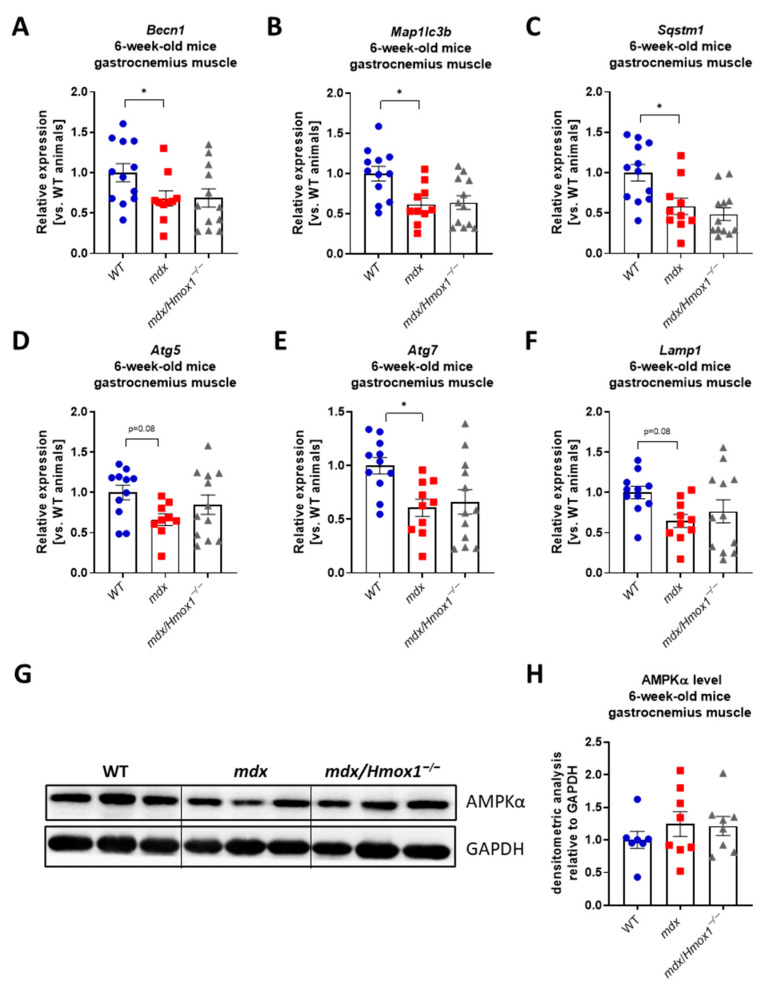
The expression of several genes regulating autophagy is decreased in gastrocnemius of 6-week-old *mdx* mice without the further influence of *Hmox1* deficiency. Analysis of mRNA levels of factors regulating autophagy in gastrocnemius muscle of WT, *mdx*, and *mdx*/*Hmox1^−/−^* mice. The expression of *Becn1* (**A**), *Map1lc3b* (**B**), *Sqstm1* (**C**), *Atg5* (**D**), *Atg7* (**E**), and *Lamp1* (**F**) was analyzed by qRT-PCR; n = 9–12/group; Results presented as mean ± SEM. The protein level of AMPKα presented by Western blot (**G**) and its densitometric analysis (**H**), n = 7–8/group. GAPDH was used as a loading control. Results presented as mean ± SEM. * *p* < 0.05 by one-way ANOVA with Tukey’s post hoc test.

**Figure 3:**
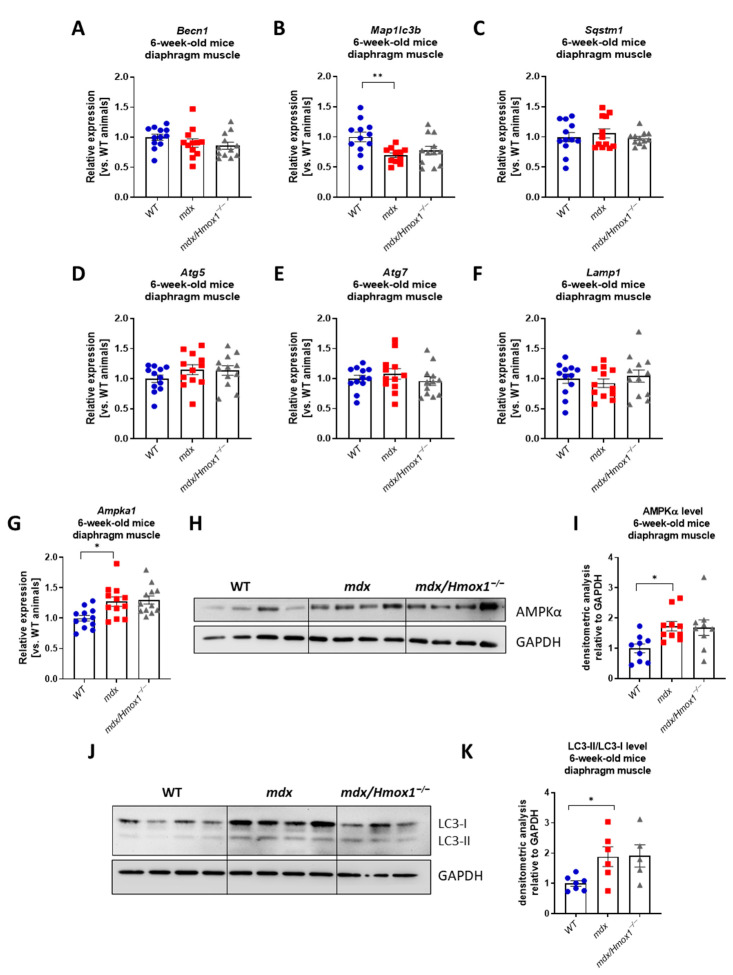
The expression of genes regulating autophagy is not changed by the additional lack of *Hmox1* in the diaphragm of 6-week-old dystrophic mice. Analysis of mRNA levels of genes regulating autophagy in diaphragm muscle of WT, *mdx*, and *mdx*/*Hmox1^−/−^* animals. The expression of *Becn1* (**A**), *Map1lc3b* (**B**), *Sqstm1* (**C**), *Atg5* (**D**), *Atg7* (**E**), *Lamp1* (**F**), and *Ampka1* (**G**) was analyzed by qRT-PCR; n = 11–12/group. The protein level of AMPKα presented as a representative Western blot (**H**) and its densitometric analysis (**I**), n = 9–10/group. GAPDH was used as a loading control. Representative Western blot analysis of LC3-I/LC3-II protein (**J**) and densitometric analysis of LC3-II/LC3-I ratio (**K**), n = 5–7/group. GAPDH was used as a loading control. Results presented as mean ± SEM. * *p* < 0.05, ** *p* < 0.01 by one-way ANOVA with Tukey’s post hoc test.

To analyze the role of autophagy in DMD progression, the panel of six markers, namely beclin-1 (*Becn1*), microtubule-associated proteins 1A/1B light chain 3 (*Map1lc3b*), sequestosome 1 (*Sqstm1*), autophagy-related gene 5 (*Atg5*), autophagy-related gene 7 (*Atg7*), and lysosomal-associated membrane protein 1 (*Lamp1*) has been evaluated in two muscles. In gastrocnemius, the mRNA level of those factors was decreased in dystrophic animals (the tendency was observed for *Atg5* and *Lamp1*) (Figure 2A–F). In the diaphragm, which is also potently affected by disease progression, no differences in the expression of genes regulating autophagy, except for the diminished *Map1lc3b* detected in dystrophic animals, were observed (Figure 3A–F). Interestingly, we did not see any differences between *mdx* and *mdx*/*Hmox1*^−/−^ mice in both analyzed muscles (Figure 2A–F and Figure 3A–F). In the gastrocnemius muscle of dystrophic animals, the protein level of AMP-activated protein kinase (AMPKα), a regulator of the autophagy-mitophagy pathway [27], was not significantly affected (Figure 2G,H). On the other hand, the upregulation of the AMPKα mRNA and protein was evident in the diaphragm (Figure 3G–I).

However, the effect of HO-1 deficiency was observed neither in gastrocnemius nor in the diaphragm. Finally, although *Map1lc3b* expression was not changed, the protein levels of LC3s (encoded by this gene) were upregulated in the dystrophic diaphragm (Figure 3J). The ratio of LC3-II/LC3-I proteins was significantly increased in *mdx* mice in comparison to WT (Figure 3K) but the lack of HO-1 did not affect the level of the aforementioned proteins (Figure 3J–K).

### 2.3. 6-Week-Old Mdx Mice Exhibit Mitochondrial Dysfunction and Decreased Expression of the Genes Regulating Mitophagy

**Figure 4:**
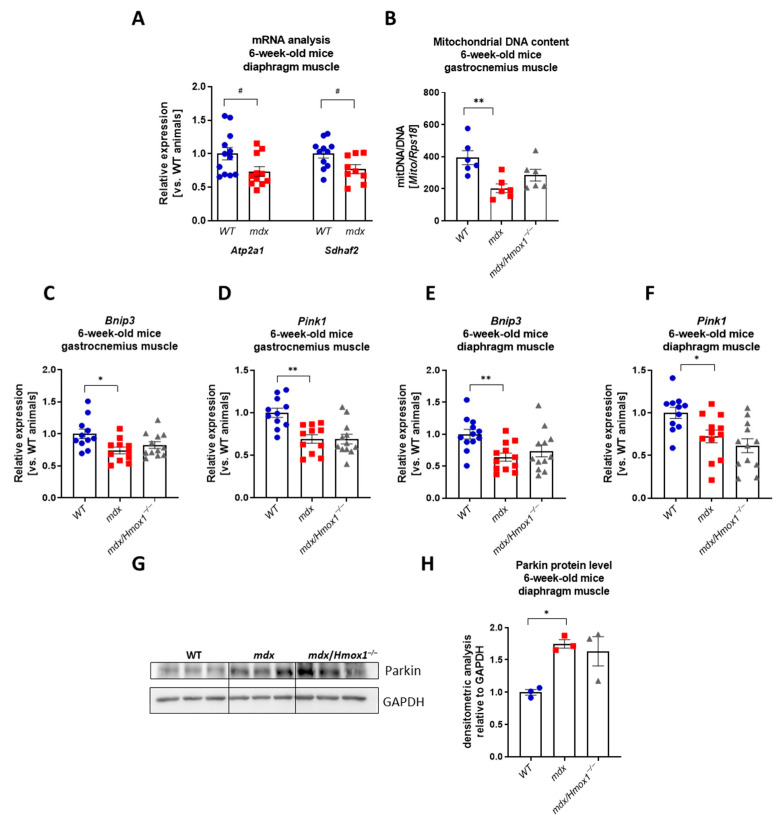
Genes and proteins regulating mitochondrial homeostasis in 6-week-old mice. mRNA level of *Atp2a1* and *Sdhaf2* in 6-week-old WT and *mdx* mice analyzed by qRT-PCR (**A**); n = 9–12/group. Decreased mitochondrial DNA content in *mdx* mice (**B**), qRT-PCR, n = 6/group. Analysis of the mRNA level of *Bnip3* and *Pink1* genes regulating mitophagy in gastrocnemius (**C**,**D**) and diaphragm (**E**,**F**) muscles from WT, *mdx*, and *mdx*/*Hmox1^−/−^* genotypes, qRT-PCR; n = 11–12/group. Western blot analysis of Parkin protein (**G**) and densitometric analysis of Parkin level in diaphragm muscle (**H**), n = 3/group. GAPDH was used as a loading control. Results presented as mean ± SEM. # *p* < 0.05 with Student’s *t*-test * *p* < 0.05, ** *p* < 0.01 by one-way ANOVA with Tukey’s post hoc test.

Mitochondrial dysfunction is a pathological feature of DMD [28]. Knowing that mitochondria are a major source of cellular ATP involved in Ca^2+^ regulation and apoptotic signaling, we analyzed the expression of ATPase sarcoplasmic/endoplasmic reticulum Ca^2+^ transporting 1 (*ATP2a1*) regulating calcium metabolism and succinate dehydrogenase complex assembly factor 2 (*Sdhaf2*), encoding protein building mitochondrial complex II. A statistically significant decrease in their expression (Figure 4A) in the dystrophic diaphragm might indicate changes in mitochondrial signaling and homeostasis. Concomitantly, mitochondrial DNA content was decreased in *mdx* mice (Figure 4B). Therefore, we aimed to characterize mitophagy, the process responsible for the degradation of damaged or dysfunctional mitochondria [6]. The expression level of the Bcl2/adenovirus E1B 19 kDa protein-interacting protein 3 (*Bnip3*) was decreased in *mdx* mice both in the gastrocnemius and diaphragm (Figure 4C,E). However, no differences in the level of the *Bnip3* transcript between *mdx*/*Hmox1*^−/−^ and *mdx* individuals were found. Moreover, when PTEN-induced putative kinase 1 (*Pink1*) mRNA level was evaluated, we have found a very similar pattern of the expression. Lack of dystrophin resulted in a decreased level of *Pink1* transcript in 6-week-old *mdx* mice, in both analyzed muscles. Nevertheless, HO-1 deficiency did not affect *Pink1* expression in the gastrocnemius and diaphragm of 6-week-old mice (Figure 4D,F). Concomitantly with the previous findings for autophagy-related markers, the protein level of Parkin was induced in diaphragm (Figure 4G,H) of 6-week-old dystrophic animals, without any effect of HO-1 depletion. The opposite regulation of those mitophagy-related factors on mRNA and protein level together with decreased mitochondrial DNA content in dystrophic animals indicates the high complexity of this process and emphasizes the necessity of further experiments.

### 2.4. The Lack of HO-1 Does Not Affect Mitophagic and Autophagic Genes Expression in Older mdx Mice

**Figure 5:**
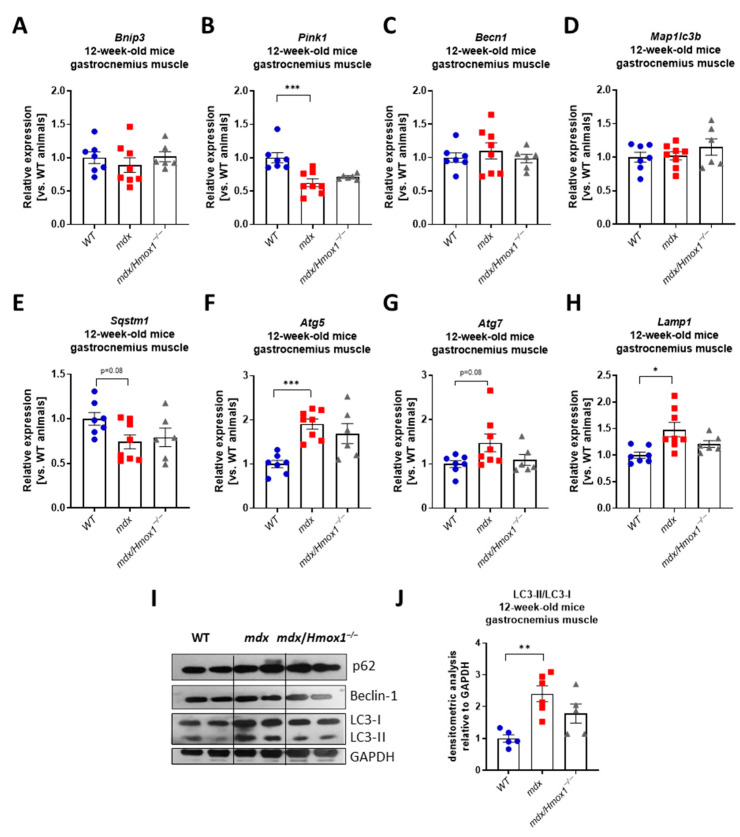
Expression of genes regulating mitophagy and autophagy in 12-week-old WT, *mdx*, and *mdx*/*Hmox1^−/−^* mice. Analysis of mRNA level of genes regulating autophagy in gastrocnemius muscle. The expression of *Bnip3* (**A**), *Pink1* (**B**), *Becn1* (**C**), *Map1lc3b* (**D**), *Sqstm1* (**E**), *Atg5* (**F**), *Atg7* (**G**), and *Lamp1* (**H**) was analyzed by qRT-PCR; n = 6–8/group. Western blot analysis of p62, Beclin-1, LC3-I, and LC3-II level (**I**) and densitometric analysis of LC3-II/LC3-I ratio (**J**), n = 5–6/group. GAPDH was used as a loading control. Results presented as mean ± SEM. * *p* < 0.05, ** *p* < 0.01, *** *p* < 0.005 by one-way ANOVA with Tukey’s post hoc test.

To check if the lack of the effect of *Hmox1* deficiency observed in our analysis might be the result of the relatively young age of the mice used in this study, we decided to perform a similar analysis in older, 12-week-old animals. We have concentrated on gastrocnemius, as we observed prominent changes in this muscle in 6-week-old *mdx* mice. Expression of *Bnip3, Becn1*, and *Map1lc3b* did not differ between WT and *mdx* animals whereas the expression of *Pink1* and *Sqstm1* was diminished in dystrophic muscle, similarly to 6-week-old mice (Figure 5A–E). Interestingly, an increase in the expression of *Atg5*, *Atg7*, and *Lamp1* was found (Figure 5F–H). Although changes in p62 and Beclin-1 protein levels were rather moderate, a prominent rise in LC3-II/LC3-I proteins ratio was noticed when comparing WT and *mdx* mice (Figure 5I,J). Again, the lack of *Hmox1* expression did not affect greatly the mRNA and protein level of analyzed mitophagy and autophagy markers (Figure 5A–J).

## 3. Discussion

DMD, a disease resulting in progressive muscle weakness is caused by a mutation in the *Dmd* gene leading to the loss of dystrophin protein [23]. Although the cause of the disease has been known for a long time, no effective drug has been found yet. Therefore, numerous studies are still being conducted, mostly using the animal model of DMD—*mdx* mice. [29], which were also utilized in the present study. In our hands, *mdx* mice already at 6 weeks of age demonstrated changes in the expression of disease markers, like increased levels of OPN, MMP-9, and PAI-1, but more importantly, they exerted decreased muscle functionality as assessed by grip strength assay.

Literature data on autophagy disturbances in the *mdx* mice and DMD patients are inconclusive. Some of them suggest autophagic flux to be insufficient, and that reactivation of autophagy improves muscle function and reduces muscle damage [10]. Pauly et al. demonstrated that pharmacological activation of AMPK triggered autophagy in dystrophic mice, leading to the removal of defective mitochondria and improvement of diaphragm structure and function [9]. On the other hand, several studies have shown activation of the autophagy in dystrophic muscles of *mdx* animals as well as in individuals with DMD, but this process may be greatly affected by age and disease progression [17,30]. Nevertheless, to check if the disturbed expression of genes regulating mitophagy and autophagy may be further affected by the factors known to modulate DMD pathology, we concentrated on assessing the possible role of cytoprotective, anti-oxidant, and anti-inflammatory HO-1 in this regulation.

In our previous study, we observed that around 3-month-old double knockout animals lacking dystrophin and *Hmox1* demonstrate impaired exercise capacity and aggravated DMD pathology in comparison to *mdx* animals [19]. Our results showed an increased level of both CK and LDH activity in 6- and 12-week-old *mdx*/*Hmox1^−/−^* animals, together with prominently alleviated inflammatory cell infiltration. However, when more in-depth analysis of M1-like and M2-like subpopulations of macrophages was performed, the lack of *Hmox1* did not result in any significant changes. In the present study, we found an increase in the expression of some profibrotic markers and, similarly to our previous work, the level of tissue damage markers in serum of 12-week-old mice, such as LDH and CK activity. Moreover, a higher percentage of granulocytes and monocytes at the expense of lymphocytes within WBC was noticed already in 6-week-old *mdx/Hmox1^−/−^* in comparison to *mdx* animals. Interestingly, we have previously shown that similar changes could be observed in the 8-week-old dystrophic animals treated with the pharmacological inhibitor of HO-1 activity, SnPPIX [19]. Of note, in such relatively young animals, the diminished expression of both mitophagy and autophagy-regulating genes in the gastrocnemius muscle of *mdx* mice was evident. Interestingly, we did not find prominent changes in the expression of those genes in the diaphragm, except the downregulation of *Map1lc3b*, a ubiquitin-like modifier involved in the formation of autophagosomal vacuoles (autophagosomes), suggesting the muscle-specific effects. Moreover, *Hmox1* deficiency did not affect the expression of the analyzed factors. Even in the older animals, no significant exacerbation of this phenomenon was observed in dystrophic mice deficient in *Hmox1*. Interestingly, when the autophagy regulators were assessed on the protein level, upregulation of AMPKα in the dystrophic diaphragm and LC3 proteins both in diaphragm and gastrocnemius muscle was evident

Several different molecular mechanisms of mitophagy have been described [31]. The most frequently depicted is based on the Pink1/Parkin interaction [32]. Kang et al. demonstrated the accumulation of damaged mitochondria in the myocardium of *mdx* mice caused by disturbances in mitophagy through decreased expression of *Pink1* and *Parkin* [12], which stays in line with the results obtained in the present study in the gastrocnemius and diaphragm muscles of 6-week-old animals. However, the protein level of Parkin was higher in dystrophic tissues than in WT mice suggesting the posttranscriptional regulation. Moreover, in our study, we confirmed decreased mitochondrial DNA content in dystrophic animals [17], which could be correlated with higher Parkin protein level, as its overexpression was shown to induce mitochondrial removal from the cells [33]. Nevertheless, no effect of *Hmox1* deletion on mitochondrial DNA content was noticed when compared to *mdx* mice.

Another component of mitophagy machinery is BNIP3, a member of a subfamily of death-inducing mitochondrial proteins, present on the outer mitochondrial membrane (OMM) and interacting with the autophagosome-localized LC3 protein [34]. Similar to our findings, De Palma et al. reported lower *Bnip3* expression in the tibialis anterior muscle and diaphragm isolated from 4-month-old *mdx* mice [10] and Sebori et al. found reduced expression of this gene in the quadriceps of even older, 22-week-old dystrophic mice [11]. According to disturbances in mitophagy markers, we also confirmed, previously described in the literature [35], decreased levels of *Atp2a1*, encoding the SERCA1 pump responsible for Ca^2+^ removal from the cytosol into the lumen of the sarcoplasmic reticulum following muscular contraction. Dystrophic muscle with impaired Ca^2+^ handling and increased sarcoplasmic Ca^2+^ levels are prone to myofiber death through Ca^2+^-dependent protease activation and mitochondrial Ca^2+^ overload in dystrophic muscle. Moreover, a reduced level of *Sdhaf2* indicating impaired tissue energetics and mitochondrial function was also found in our analysis of *mdx* mice.

We noted a decrease in the expression of *Atg5*, *Atg7*, *Becn1*, *Map1lc3b*, *Lamp1*, and *Sqstm1* genes in the gastrocnemius muscle, but in the diaphragm of 6-week-old mice, only *Map1lc3b* was reduced. Similarly to our study, Panza et al. reported diminished expression of among others, *Lamp1, Becn1*, and *Atg7*, in quadriceps of 7-week-old animals [16] and the expression of *Becn1, Map1lc3b, Lamp1*, and *Sqstm1* was decreased in the same muscle of 22-week-old mice [11]. No difference in the mRNA levels of the *Map1lc3a*, *Map1lc3b*, and *Sqstm1* genes was found between WT and *mdx* mice hearts [8]. In our hands, in older, 12-week-old animals, the pattern of analyzed gene expression was complex with down-regulation of *Pink1* and *Sqstm1* and up-regulation of *Atg5*, *Atg7*, and *Lamp1*. Although the protein level of p62 and Beclin-1 (encoded by *Sqstm1* and *Becn1*, respectively) was not greatly affected by the lack of the dystrophin, the upregulation in the LC3s (encoded by *Map1lc3b*) protein level and the ratio of LC3-II/LC3-I was noticed. Such discrepancies in mRNA and protein levels could be explained by the multi-stage regulation of this process, and they may indicate the complexity of autophagy regulation in DMD. For example, despite the upregulation of several autophagy-related proteins that could suggest induction of the self-degradation machinery, increased p62 protein is often correlated with disturbed autophagy [36,37]. The inconsistency of the published results regarding the status of autophagy/mitophagy in DMD may result also from additional factors, including the age of animals or type of analyzed muscles (which may greatly differ in the metabolic state). Some discrepancies may be also the cause of various genetic backgrounds of animals used in different studies (e.g., in this study, animals with a mixed genetic background C57BL/10ScSn/J × C57BL/6xFVB were used, whereas e.g., Sebori et al. [11] analyzed C57BL/10 (WT) and C57BL/10ScSn-*Dmd^mdx^*/J (*mdx*) strains. Moreover, it has been shown that mRNA levels may be increased when autophagy is impaired, and mRNA transcripts may be stable when the autophagic flux is active, proving that transcriptional regulation of autophagy does not always closely correlate with functional autophagic flux [38]. Furthermore, such discrepancies could be related to the used methodology, as a measurement of autophagic flux in tissue samples is challenging [38]. For example, a higher level of LC3-II might indicate enhanced autophagosome synthesis or, among others, reduced autophagosome turnover [39]. Caution during interpretation should be advised, especially as western blots show only snapshots in time. For proper interpretation of the data, usage of autophagy inductors or inhibitors should be applied to measure autophagy flux; however, its utilization in the animal models remains to be difficult and prone to misinterpretation. In conclusion, whether autophagy/mitophagy is beneficial or harmful to dystrophic skeletal muscle may depend on its size and the specific context in which it occurs.

The main aim of the performed study was to check if the disturbed expression of genes regulating mitophagy and autophagy may be affected by cytoprotective, anti-oxidant, and anti-inflammatory HO-1. Although we have previously found HO-1 deficiency as the condition accelerating inflammation, fibrosis, and muscle damage in *mdx* mice [19], in the present work we did not show any effect of *Hmox1* absence on the mRNA and protein level of mitophagy/autophagy factors. Until now, no data regarding the possible role in the control of these pathways by HO-1 in DMD has been published; however, such effects in other disease models were published. Unuma et al. showed that induction of autophagy by HO-1 in rats treated with lipopolysaccharide (LPS) promotes the removal of damaged mitochondria in hepatocytes, thereby reducing oxidative stress and preventing sepsis-induced liver damage [40]. Moreover, Zhao et al. found that HO-1 counteracts oxidative stress and inflammation, and enhances autophagy in the myocardium to prevent its damage in diabetic cardiomyopathy [41]. In turn, mice lacking the *Hmox1* gene in cardiomyocytes showed abnormalities related to the dysregulation of the Pink1/Parkin pathway [42]. Nevertheless, we cannot exclude the possibility, that such a strong effect of dystrophic phenotype on the autophagy and mitophagy homeostasis, observed by us and others [10], masks any influence of *Hmox1* deletion in the *mdx* mouse model. It would be of interest to investigate, whether chemical upregulation of HO-1 and/or genetic *Hmox1* overexpression, could affect dystrophy-related alterations in these processes.

Even though we did not confirm our hypothesis that “self-eating machinery” is impaired in *Hmox1*-lacking dystrophic animals, it cannot be excluded that such a situation might be present in DMD patients. HO-1 level and its activity vary in the human population, as a consequence of *Hmox1* promoter polymorphism. Although not studied in DMD patients, it is well known that variations in the number of (GT)n repeats affect the cytoprotective, anti-inflammatory, and pro-angiogenic functions of HO-1 and may influence the progression of various diseases [43]. More studies in DMD individuals or human induced pluripotent stem cells, obtained from dystrophic boys with different HO-1 levels, and differentiated to, e.g., skeletal muscle cells or cardiomyocytes, are required to fully answer this question.

In summary, our experiments did not confirm the role of HO-1 in the regulation of the autophagy and mitophagy markers in DMD as postulated in other diseases. It might be suggested that lack of dystrophin disturbs autophagy to such an extent, which is not affected more by the additional lack of HO-1, which, nevertheless, aggravates other conditions in *mdx* animals. Although more detailed experiments are required, especially analyzing the autophagic flux in vivo and a more detailed examination of the mitochondrial structure and number, our study suggests disturbances in mitochondrial metabolism and mitophagy already in 6-week-old *mdx* mice. Those results may help to better understand the pathology of DMD and enable the development of effective therapy improving the length and quality of life of patients with this incurable disease.

## 4. Materials and Methods

### 4.1. Animal Models

The study was approved by the 2nd Institutional Animal Care and Use Committee (IACUC) in Kraków, Poland (approval number: 323/2018). Both *mdx* (C57BL/10ScSn-*Dmd^mdx^*/J) and control (C57BL/10ScSnJ) mice were purchased from the Jackson Laboratory whereas HO-1 knockout animals were generated by breeding of *Hmox1*^−/+^ mice at C57BL/6×FVB background originally gifted by Dr. Anupam Agarwal, Birmingham, USA. Crossing animals leading to the delivery of double knockouts on C57BL/10ScSn and C57BL/6xFVB mixed background (mice lacking dystrophin and HO-1, *mdx*/*Hmox1*^−/−^) was done as described previously [19]. Mice genotyping was performed by PCR method using DNA isolated by Genomic mini kit (A&A Biotechnology, Gdańsk, Poland) from mouse tails. Animals were housed in specific pathogen-free conditions with water and food available *ad libitum.* 6-week-old and 12-week-old male littermates or age-matched wild type (WT), *mdx*, and *mdx*/*Hmox1*^−/−^ (all on C57BL/10ScSn and C57BL/6xFVB mixed background) mice were used for experiments.

### 4.2. Grip Strength Assay

Muscular strength was assessed in 6-week-old mice as previously described [44] using a triangular pull bar (Ugo Basile SRL, Gemonio (VA) Italy). Briefly, mice were allowed to grasp the grid using forelimbs and after moving toward the bar, they were pulled back horizontally until the grip was released. Results were calculated as the mean of five measurements (carried out with a 1-min break between measurements), normalized to body weight, and expressed as gf/g body weight (gf/g BW).

### 4.3. Total Blood Cell Count

Blood collected directly from *vena cava* to the EDTA-coated tubes was analyzed using scil Vet ABC™ Hematology Analyzer (HORIBA ABX, Warszawa, Poland). The number of white blood cells (WBC, 10^3^/µL), as well as the percentage of lymphocytes, granulocytes, and monocytes, among WBC, was determined.

### 4.4. Analysis of Gene Expression by Real-Time Quantitative PCR (qRT-PCR)

Total RNA was isolated from the gastrocnemius and diaphragm muscles by using the modified Chomczynski method [45] with QIAzol (Qiagen, Hilden, Germany) as the lysis reagent. Tissues were homogenized using TissueLyser (Qiagen, Hilden, Germany) and then subjected to chloroform extraction and isopropanol precipitation. The quality and concentration of RNA were determined spectrophotometrically (NanoDrop, Thermo Fisher Scientific, Waltham, MA, USA). Moreover, 1 µg of RNA was reverse-transcribed into cDNA using RevertAid Reverse Transcriptase (Thermo Fisher Scientific, Waltham, MA, USA), and qRT-PCR reactions were performed using SYBR Green JumpStart Taq Ready Mix (Sigma-Aldrich, St. Louis, MO, USA) and specific primers (listed in Table 1) in StepOnePlus real-time PCR system (Thermo Fisher Scientific, Waltham, MA, USA). *Eef2* served as a housekeeping gene. For each sample, the mRNA levels of the gene of interest were normalized to *Eef2* based on the comparative C_t_ method using the equation 2^−ΔCt^ (ΔC_t_ = C_t gene of interest_ − C_t *Eef2*_) and presented as the relative expression in comparison to WT animals.

### 4.5. Mitochondrial DNA Content

DNA content measurement was performed by real-time quantitative PCR using SYBR Green JumpStart Taq Ready Mix (Sigma-Aldrich, St. Louis, MO, USA) and specific primers (listed in Table 2, based on [46]). Ribosomal protein S18 (*Rps18*) was used for normalization. DNA isolation from gastrocnemius muscle was performed using a Genomic mini kit (A&A Biotechnology, Gdańsk, Poland). Results are presented as the relative expression in comparison to WT animals.

### 4.6. Proteome Profiler Array

Diaphragm tissues were disrupted using a tissue homogenizer (TissueLyser, Qiagen, Hilden, Germany). The protein content was determined by bicinchoninic acid (BCA, Sigma-Aldrich, St. Louis, MO, USA) assay, and 100 µg of total protein was used for proteome profiling using Proteome Profiler™ array according to the manufacture’s protocol (R&D Systems, Minneapolis, MN, USA).

### 4.7. CK and LDH Activity Assessment

For the assays, blood was collected from the *vena cava*. Clear, non-hemolyzed serum was prepared by allowing the blood to clot at room temperature for 30 min and then centrifuged at 2000× *g* for 10 min at 4 °C. The activity of CK and LDH was measured in serum using the diagnostic Liquick Cor-CK and Liquick Cor-LDH kits, respectively, according to the vendor’s instruction (Cormay, Warsaw, Poland). The absorbance values obtained in 10 times diluted serum were then converted to LDH or CK activity (U/l) using the formula supplied with the kit.

### 4.8. ELISA Assay

OPN concentration in 750 times diluted serum of 6- and 12-week-old animals was determined by ELISA following the vendor’s protocol (R&D Systems, Minneapolis, MN, USA, cat no. MOST00). HO-1 content in gastrocnemius muscle of 6-week-old mice was analyzed using Mouse Heme Oxygenase 1 ELISA Kit (Abcam, Cambridge, UK, cat no. ab204524). The results measured as ng/mL are shown as the percentage of WT animals.

### 4.9. Western Blot

Fragments of muscles (gastrocnemius/diaphragm) were homogenized using an automatic TissueLyser (Qiagen, Hilden, Germany) in ice-cold PBS containing 1% Triton X-100 and protease inhibitors (1 μg/mL phenylmethylsulfonyl fluoride, 1 μg/mL leupeptin, and 1 μg/mL aprotinin). Total protein concentration was determined by the bicinchoninic acid protein assay kit (Sigma-Aldrich, St. Louis, MO, USA), according to the vendor’s protocol. Protein lysates were subjected to western blot as described previously [47]. The primary antibodies used were rabbit anti-LC3-I/LC3-II, rabbit anti-p62, rabbit anti-Beclin-1, mouse anti-Parkin, rabbit anti-AMPKα, and mouse anti-GAPDH (listed in Table 3). Appropriate peroxidase-conjugated secondary antibodies (goat anti-mouse 1:20000; BD Pharmingen, San Diego, CA, USA and goat anti-rabbit, 1:5000, Cell Signaling Technology, Danvers, MA, USA) were used to detect the proteins of interest by chemiluminescence (Immobilon Western Chemiluminescent HRP Substrate, Millipore, Burlington, MA, USA).

### 4.10. Statistical Analyses

Data are presented as mean ± SEM. Differences between groups were tested for statistical significance using the one-way ANOVA followed by Tukey’s post hoc test when more than two groups were analyzed or the unpaired 2-tailed Student’s *t*-test for the comparison of two groups (Figure 4A) *p* < 0.05 was considered significant. The outliers were identified based on Grubb’s test.

## Figures and Tables

**Table 1:** The sequences of primers used for the determination of gene expression on mRNA level by qRT-PCR.

Gene	Full Gene Name	Primer Sequence
*Ampka1*	AMP-activated protein kinase	F: 5′-TGTGACAAGCACATTTTCCAA-3′
		R: 5′-CCGATCTCTGTGGAGTAGCA-3′
*Atg5*	Autophagy-related gene 5	F: 5′-CTGAAGATGGAGAGAAGAGG-3′
		R: 5′-GGGGACAATGCTAATATGAAG-3′
*Atg7*	Autophagy-related gene 7	F: 5′-CTGTTCACCCAAAGTTCTTG-3′
		R: 5′-TCTAAGAAGGAATGTGAGGAG-3′
*Becn1*	Beclin-1	F: 5′-CAATAATTTCAGACTGGGTCG-3′
		R: 5′-ATTTGTCTGTCAGAGACTCC-3′
*Bnip3*	Bcl2/adenovirus E1B-19 kDa interacting protein 3	F: 5′-ACCACAAGATACCAACAGAG-3′
		R: 5′-AATCTTCCTCAGACAGAGTG-3′
*Eef2*	Elongation factor-2	F: 5′-AGAACATATTATTGCTGGCG-3′
		R: 5′-CAACAGGGTCAGATTTCTTG-3′
*Hmox1*	Heme oxygenase-1	F: 5′-CCTCACTGGCAGGAAATCATC-3′
		R: 5′-CCTCGTGGAGACGCTTTACATA-3′
*Lamp1*	Lysosomal-associated membrane protein 1	F: 5′-ATTGCAGTTTGGGATGAATG-3′
		R: 5′-TTGCACTTGTATGAGTTTCC-3′
*Map1lc3b*	Microtubule-associated proteins 1A/1B light chain 3	F: 5′-GCTCATCAAGATAATCAGACG-3′
		R: 5′-GCATAAACCATGTACAGGAAG-3′
*Pink1*	PTEN-induced kinase 1	F: 5′-ACTTACAGAAGATCCAGAGATG-3′
		R: 5′-CTTCATAACGAGGAACAGTG-3′
*Spp1*	Secreted phosphoprotein 1	F: 5′-CCATCTCAGAAGCAGAATCTCCTT-3′
		R: 5′-GGTCATGGCTTTCATTGGAATT-3′
*Sqstm1*	Sequestosome 1	F: 5′-AATGTGATCTGTGATGGTTG-3′
		R: 5′-GAGAGAAGCTATCAGAGAGG-3′

**Table 2:** The sequences of primers used for the determination of mitochondrial DNA content by qRT-PCR.

	Primer Sequence
*Mito* (mitochondrial)	F: 5′-CTAGAAACCCCGAAACCAAA-3′
	R: 5′-CCAGCTATCACCAAGCTCGT-3′
*Rps18* (genomic)	F: 5′-TGTGTTAGGGGACTGGTGGACA-3′
	R: 5′-CATCACCCACTTACCCCCAAAA-3′

**Table 3:** List of primary antibodies used for western blot.

Antibody	Company	Catalog Number	Dilution
AMPKα	Cell Signaling Technology Danvers, MA, USA	2603	1:500
Beclin-1	Cell Signaling Technology Danvers, MA, USA	3495	1:500
GAPDH	Santa Cruz Biotechnology Dallas TX, USA	sc-59540	1:1000
LC3-I/II	Cell Signaling Technology Danvers, MA, USA	12741	1:500
Parkin	Cell Signaling Technology Danvers, MA, USA	4211	1:500
SQSTM1/p62	Cell Signaling Technology Danvers, MA, USA	39749	1:500

## Data Availability

Not applicable.
